# The Codesign, Development and Implementation of an Indian Poststroke Swallow and Hydration Care Bundle: A Feasibility Study

**DOI:** 10.1155/srat/9745360

**Published:** 2026-07-31

**Authors:** Elizabeth Boaden, C. Elizabeth Lightbody, Stephanie P. Jones, Colette Miller, Dominique A. Cadilhac, Sandy Middleton, Gordon Prescott, Chris Sutton, Jeyaraj D. Pandian, M. V. Padma Srivastava, P. N. Sylaja, Caroline L. Watkins

**Affiliations:** ^1^ School of Nursing and Midwifery, University of Lancashire, Preston, UK, deakin.edu.au; ^2^ Stroke and Ageing Research, School of Clinical Sciences, Monash University, Clayton, Australia, monash.ac.za; ^3^ Nursing Research Institute, St Vincent′s Health Network Sydney and Australian Catholic University, Sydney, Australia; ^4^ Lancashire Clinical Trials Unit, University of Lancashire, Preston, UK; ^5^ Department of Neurology, Christian Medical College and Hospital, Neurology, Ludhiana, India, cmch-vellore.edu; ^6^ Chairperson of Neurology at Paras Hospitals Gurugram, Gurugram, India; ^7^ Department of Neurology, Sree Chitra Tirunal Institute for Medical Sciences and Technology, Neurology, Thiruvananthapuram, India, sctimst.ac.in; ^8^ School of Nursing and Midwifery, University of Central Lancashire, Preston, UK, uclan.ac.uk

**Keywords:** dysphagia, evaluation, hydration, implementation, India, low- and middle-income countries, nurses, stroke, swallowing

## Abstract

**Background:**

Stroke is increasing in low‐ and middle‐income countries. Stroke unit care, including evidence‐based practice such as swallowing and hydration protocols, is associated with reduced disability and mortality.

**Objectives:**

The aim of this study is to codesign a swallowing and hydration care bundle for nurses to use with acute stroke patients, and to implement and evaluate the bundle on three acute stroke units across India.

**Methods:**

A multicentre, preimplementation and postimplementation feasibility study was conducted. We codesigned a care bundle (with stakeholders) which included the Global Evaluation of Swallowing and hydration screening and related actions/management. Implementation used clinical champions, education and training, barrier and enabler identification and site support. Participants included consecutively admitted patients, aged ≥ 18 years, with a clinical diagnosis of acute stroke and a persistent neurological deficit on presentation, who were admitted to a study site within 2 weeks of stroke onset.

**Results:**

Fifty‐three healthcare staff completed the care bundle training. We recruited 183 participants, preimplementation group (*n* = 92). Swallowing evaluations using different consistencies increased from 22 (23.9%) to 51 (56%). Calculated osmolarity (cOsm) increased from 0 to 66 (73%) participants. Median cOsm was 288 mmol/L (IQR: 280–295 mmol/L); 15 (23%) participants were dehydrated.

**Conclusion:**

Care bundle implementation was feasible and improved swallowing and hydration management in three sites in India.

## 1. Introduction

Stroke is the second leading cause of global disease burden, and the incidence is increasing in low‐ and middle‐income countries (LMICs) [1]. Together with cardiovascular disease, stroke is the most common cause of death and is recognised as being at epidemic proportions in LMICs [2]. In India, crude incidence of stroke ranges from 108 to 172/100,000 people per year, crude prevalence from 26 to 757/100,000 people per year and 1‐month case fatality rates from 18% to 42% [3].

Stroke unit care, including an organised specialist multidisciplinary team, is associated with reduced mortality [4]. A stroke workforce with enhanced knowledge and skills, combined with stroke unit care quality indicators, can be improved via structured interventions such as swallowing and hydration [5]. However, in India, specialist stroke and rehabilitation centres are limited, and few healthcare staff have specialist stroke training [6]. Swallowing difficulties (dysphagia) are common following stroke and are associated with malnutrition, dehydration and aspiration pneumonia [7], with concomitant increases in mortality and morbidity [8]. A UK sentinel stroke audit [9] showed that undertaking a swallow evaluation and provision of adequate fluid and nutrition are associated with lower 30‐day mortality.

Approximately 50% of acute stroke patients have swallowing difficulties [10]. Vigilance is required by healthcare staff in the identification, evaluation and management of swallowing difficulties, as dysfunction fluctuates on a 24‐h basis with both resolving and emerging symptoms. In India, a common approach to swallowing screening is a water swallow test (WST), but swallow physiology alters according to the texture of food and drink, with mixed textures and liquids being more difficult to control and more likely to be aspirated into the airway [11]. A failed WST results in people being placed nil orally and referred for a detailed evaluation and clinically assisted nutrition and hydration. A passed WST results in people taking food and drinks that have not been assessed. A study that used flexible endoscopic evaluation of swallowing on people who failed the WST found that 56% of people (1151) could safely swallow a normal diet and 25% could safely consume a puree diet [12]. Swallow screening tools that consider a limited range of textures may not be commensurate with the International Dysphagia Diet Standardisation Initiative (IDDSI) [13, 14] and have not been assessed within LMICs.

Although the National Indian Guidelines for Stroke [15] recognise dysphagia and dehydration as significant complications following stroke, there is a paucity of research evidence regarding swallowing and hydration assessment and management in stroke units in India [16]. Prior to the present study, routine swallow screening and systematic assessment of hydration were not consistently implemented for all stroke patients in the stroke units in India. Where swallow screening was undertaken, practices varied considerably; screening was primarily undertaken by doctors who used the gag reflex, a WST or a swallow test with limited consistencies that were not commensurate with IDDSI. Access to comprehensive swallowing and hydration assessment was often limited.

Consequently, patients with stroke were frequently discharged within days of admission, often with nasogastric tubes and no robust management or process for a swallow review.

A common sequela of dysphagia poststroke is dehydration. Direct measurement of serum/plasma osmolality (pOsm) is the gold standard for determining dehydration; raised pOsm usually indicates dehydration > 300 and ≤ 280 mOsm/kg classifies a person as hyper or hypo‐osmolar [17].

A Cochrane review [18] of impending and current water loss in older people reported a paucity of evidence that any symptom or test, including many that clinicians routinely use, has any diagnostic utility for dehydration. Another Cochrane review [19] found no evidence to guide the best volume, duration or mode of parenteral fluid delivery for people with acute stroke. Intravenous and subcutaneous fluid infusions are commonly used but may cause adverse effects, such as cannula site infection, phlebitis, infiltration, pain or swelling, and significant discomfort [20]. Nasogastric intubation is associated with pneumonia, tissue injury and local or abdominal discomfort.

The aim of this study was to codesign, codevelop, and assess the feasibility of delivery of a swallow and hydration care bundle within three sites in India.

## 2. Materials and Methods

A multicentre, preimplementation and postimplementation feasibility study using the principles of experience‐based codesign to develop a culturally, context‐specific swallowing and hydration care bundle. Senior nurses from the stroke wards were trained to become ‘clinical champions’ and use the ‘train the trainer’ approach with supported implementation.

### 2.1. Setting

Three hospital sites in India were included as a convenience sample: Christian Medical College and Hospital, Ludhiana; All India Institutes of Medical Sciences, New Delhi; and Sree Chitra Tirunal Institute for Medical Sciences and Technology, Thiruvananthapuram.

### 2.2. Subjects and Sampling

Inclusion criteria were consecutively admitted stroke patients: aged ≥ 18 years; with a clinical diagnosis of acute stroke (ischaemic stroke or primary intracerebral haemorrhage); a persistent neurological deficit on presentation; and admitted to hospital within 2 weeks of stroke onset.

Excluded patients included people with stroke that were not admitted to the stroke unit; transient ischaemic attack; or had no legally acceptable representative to consent.

### 2.3. Codesign and Development of the Care Bundle

The principles of codesign were embedded into the design, implementation and evaluation of the care bundle. Experience‐based codesign methods [21] including stakeholder engagement were used in discussion groups to develop the components of the care bundle. Facilitated by the UK team, discussion groups commenced on March 2018 at three participating hospital sites, involving 33 healthcare professionals (senior nurses, therapists and research staff).

UK and Indian expert dysphagia therapists, nurses and researchers codesigned the Global Evaluation of Swallowing (GEoS). GEoS was developed iteratively until face (items are sensible, appropriate and relevant to the people who use the measure on a daily basis) and content validities (the items comprehensively consider the different components of health to be measured) [22] were achieved. It was translated into regionally specific languages: Malayalam, Hindi and Urdu.

GEoS adopts a stepwise process of texture evaluation and volumes, comprising a check sheet to identify swallow pathophysiology, directions to guide use of the tool, instructions when to terminate the evaluation and immediate recommendations and management advice. GEoS assesses patient physiological function (including levels of alertness, positioning, voice quality and cough); a swallowing evaluation of IDDSI compliant oral intake; recommendations of IDDSI textures. GEoS allows culturally and regionally specific textures to be used as testing materials. Hospital dietetic and catering departments identified local dishes that were available within the hospital and aligned to IDDSI levels.

A hydration screening evaluation toolkit was codesigned with nurses and researchers representing each study site. The evaluation was informed by existing literature, stroke guidelines and expert knowledge from an interdisciplinary clinical‐academic writing group funded by the British and Irish Association of Stroke Physicians [23]. The tool included common methods of hydration evaluation (urea and electrolytes, urine specific gravity, urine colour, skin turgidity and calculated osmolarity), a user guide and recommended management options. Expected serum osmolarity was calculated from routinely collected point‐of‐care test data using an “osmolarity app” tailored to the Indian context and based on the Khajuria and Krahn formula [24]. The formula has been shown to be a reliable, cost‐effective and a minimally invasive predictor of directly measured serum osmolality in older adults [25]. Documentation was codesigned with the clinical champions to ensure additional clinical paperwork was clear and fit‐for‐purpose.

### 2.4. Training and Implementation

The intervention required healthcare staff to adopt additional roles and responsibilities. Preimplementation, nurses referred patients to the specialist speech and language therapist (SLT), where available, or the doctor. During the intervention, staff undertook the GEoS and hydration care bundle. We used evidence‐based implementation strategies. The project team in the United Kingdom and India ensured that permissions were embedded within the management infrastructure to support changes in practice. Using a ‘train‐the‐trainer’ approach, healthcare staff were trained to be ‘clinical champions’ to assess and manage patients′ swallowing and hydration status. Group training was delivered face‐to‐face, and staff completed online training including videos and demonstrations. Staff had facilitated discussions with the clinical champions regarding the training, implementation and logistics within sites. Competences and observations of subsequent training delivery were completed to ensure fidelity. The project team met with each site via digital platforms weekly to explore the barriers to implementation and identify solutions to enable timely resolution of issues. This supported implementation phase ensured that staff were confident to deliver ongoing training.

### 2.5. Ethics

Ethical approval was granted by the Science, Technology, Engineering and Medicine Ethics Committee at the University of Lancashire (STEM 826). Permissions were obtained from the Government of India Health Ministry Screening Committee (HMSC), the Indian Council for Medical Research (ICMR) and the Research and Development departments at each hospital site. Written/fingerprint, verbal/witnessed informed consent was obtained from all participants. The study conforms to the ethical standards set out in the Declaration of Helsinki [26].

### 2.6. Recruitment and Data Collection

Documentation was codesigned to ensure additional clinical paperwork was clear and fit‐for‐purpose. Recruitment took place between July 2018 and February 2019. Research assistants at each site completed a case report form for consecutively admitted stroke patients. Preimplementation and postimplementation data were collected on demographics (age, sex, stroke type and stroke severity), swallow screen or evaluation (in the first 3 days) and assessment of hydration (urine specific gravity, urine colour, skin turgidity, sodium, potassium, urea and calculated osmolarity [Day 2 postadmission]).

## 3. Analysis

The analysis comprised two elements: (1) evaluation of changes following implementation of the care bundle and (2) assessment of the diagnostic performance of point‐of‐care dehydration tests.

## 4. Preimplementation and Postimplementation Comparisons

Changes between the preimplementation phase and the period following introduction of the care bundle were examined. The proportion of individuals who received a swallowing evaluation and the proportion identified as dehydrated were compared across these phases. Dehydration was defined using serum osmolality, with values > 295 mOsm/kg considered indicative of dehydration (reference standard).

## 5. Accuracy of Point‐of‐Care Dehydration Tests

The diagnostic accuracy of point‐of‐care tests for dehydration (Table 1) was assessed, both individually and in combination, using laboratory serum osmolality as the reference standard.

**Table 1:** Hydration measures and their corresponding threshold values.

Measures	Normal range	Normal/hydrated	Abnormal/dehydrated
Calculated osmolarity [27]	275–295 mOsm/L	≤ 295	> 295
Sodium	135–145 mEq/L	≤ 145	> 145
Potassium	3.5–5.0 mEq/L	≤ 5.0	> 5.0
Urea	6–24 mg/dL	≤ 24	> 24
Urine specific gravity [27]	1.005–1.030	≤ 1.020	> 1.020
Urine colour [27]	Scales 1–8	1–4	5–8
Skin turgor [28]	Seconds	< 3	≥ 3

Given the nature of the study, analyses were primarily descriptive. Demographic and baseline characteristics were summarised using appropriate descriptive statistics: means and standard deviations or medians and interquartile ranges for continuous variables, and frequencies and percentages for categorical variables.

Fidelity to the swallowing evaluation protocol was assessed alongside a clinical review of reasons for deviations, informed by monitoring chart data.

For each hydration measure, values were dichotomised into normal and abnormal categories according to predefined threshold values (Table 1).

## 6. Results and Discussion

### 6.1. Results

The study aim was achieved through the successful codesign, codevelopment and implementation of a swallowing and hydration care bundle across three sites in India, demonstrating the feasibility of this approach. Fifty‐three healthcare staff who received the training successfully completed an online certificated competency assessment and three observed clinical assessments.

#### 6.1.1. Patient Demographics

A total of 183 participants were recruited to the study (92 to the preimplementation group and 91 to the postimplementation group). The date of birth for some participants (80%–90%) was unknown; for these, their ages were estimated. The median overall age was 62 years (IQR: 53, 69); 60 years (IQR: 52, 67) and 64 years (IQR: 55, 70) in the preimplementation and postimplementation groups, respectively. Almost three‐quarters of participants were male in the preimplementation group (*n* = 66, 72%) compared with 59 (65%) in the postimplementation group.

In the preimplementation group, 76 (83%) had an ischaemic stroke and 16 (17%) had a primary intracerebral haemorrhage. In the postimplementation group, 69 (76%) had an ischaemic stroke and 21 (23%) had a primary intracerebral haemorrhage.

Stroke severity is summarised in Table 2. No participants had a severe stroke. Thirty‐seven (40%) in the postimplementation group had ‘no symptoms’ or ‘minor stroke,’ compared with 24 (26%) in the preimplementation group. Mean Barthel Index scores were slightly higher in the postimplementation group (Table 1).

**Table 2:** Stroke severity and activities of daily living.

	Preimplementation (*N* = 92)	Implementation (*N* = 91)
NIHSS completed (*n* = *%*)	62 (67)	66 (73)
NIHSS score; mean (SD)	9.5 (6.4)	7.8 (6.6)
Stroke severity; *n* (%)		
0 No stroke symptoms	0	5 (8)
1–4: minor stroke	16 (26)	21 (320)
5–15: moderate stroke	34 (55)	29 (450)
16–20: moderate to severe stroke	7 (11)	5 (8)
21–42: severe stroke	5 (8)	5 (8)
Barthel Index completed (*n* = *%*)	79 (8^6^)	87 (96)
Barthel Index score; mean (SD)	6.5 (5.5)	7.1 (5.9)
Max score 20		
Barthel Index score; median (UQ, LQ)	5 (2, 10)	5 (2, 12)

#### 6.1.2. Swallowing Evaluation

The number of swallow screens undertaken decreased from 30 (32.6%) preimplementation to 14 (15%) post care bundle implementation. Conversely, the number of comprehensive swallow evaluations increased from 22 (23.9%) to 51 (56%). At Site 3, patients were referred to the SLT for a comprehensive evaluation of swallowing preimplementation. Following implementation of the care bundle, more people received a comprehensive swallowing assessment by nursing staff (Table 3).

**Table 3:** At least one swallow screen or comprehensive swallow evaluation undertaken in the first 3 days.

Swallow screens and assessments by Day 3	Preimplementation *N*(%)	Implementation *N* = (*%*)
Screen performed *n* (%)	30 (32.6)	14 (15.4)
Site 1 ^∗^	9 (39.1)	3 (13.0)
Site 2 ^∗^	7 (36.8)	3 (16.7)
Site 3 ^∗^	14 (28.0)	8 (16.0)
Comprehensive swallow evaluation *n* (%)	22 (23.9)	51 (56.0)
Site 1 ^∗^	0 (0.0)	15 (65.2)
Site 2 ^∗^	0 (0.0)	10 (55.6)
Site 3 ^∗^	22 (44.0)	26 (52.0)
Patients receiving screen and/or swallow evaluation	45 (49)	60 (66)

*Note:* Asterisk “∗” denotes percentages within the site.

#### 6.1.3. Clinical Analysis GEoS and Hydration Levels

A clinical analysis of the data explored the reasons for failing to undertake an evaluation of swallow function over the first 3 days of the implementation period. They were categorised into valid or invalid medical reasons. Valid medical reasons included: patient designated nil orally by the medical team; altered levels of consciousness rated by research assistant or doctor (i.e., at least one of two evaluations of stupor, coma and drowsy); ventilator dependent; clinical presentation such as stroke severity, severe dysarthria or dysphonia; and one patient refused an evaluation (due to severe headache). Invalid medical reasons included the patient taking oral intake following a swallow evaluation undertaken previously, taking oral intake without an evaluation or failing to have trained staff on‐rota. There were also some participants with no recorded reasons why GEos was not undertaken.

#### 6.1.4. Hydration

Preimplementation, the assessment of hydration using cOsm was not part of routine practice. Following implementation as part of the care bundle, cOsm was assessed in 66 (73%) participants. Of those, 15 (23%) participants were dehydrated, median cOsm 297 mmol/L. Median cOsm was 288 mmol/L (IQR: 280–295 mmol/L).

Postimplementation data were collected on a range of hydration measures (Table 4). Sodium, potassium and urea were measured in more than 80% of participants. Point‐of‐care tests: urine specific gravity, urine colour and skin turgor were assessed in approximately two‐thirds of participants. Based on these tests, hydration status varied from 9% to 63%, suggesting that when used in combination, these tests provide varied and often conflicting results.

**Table 4:** Hydration point‐of‐care tests and percentages above normal threshold indicating dehydration.

Measure	*N*(%)^a^	Median (IQR)	Hydrated/normal *N*(%)^b^	Dehydrated/abnormal *N*(%)^b^
Calculated osmolarity (cOSM)	66 (73)	288 (280, 295)	51 (77)	15 (23)
Sodium	76 (84)	136 (133.5, 139)	74 (97)	2 (3)
Potassium	76 (84)	3.9 (3.5, 4.3)	75 (99)	1 (1)
Urea	75 (82)	15 (11, 31)	48 (64)	27 (36)
Urine specific gravity	64 (70)	1.02 (1.015, 1.02)	54 (84)	10 (16)
Urine colour	66 (73)	3 (2, 4)	60 (91)	6 (9)
Skin turgor	65 (71)	1 (0, 1)	24 (37)	41 (63)

^a^Percentage of those in care bundle (*n* = 91).

^b^Percentage of those for whom the measure was used.

## 7. Discussion

To our knowledge, this is the first study to codesign an innovative, context‐specific swallowing and hydration care bundle for patients with stroke delivered by the healthcare team in India. The care bundle demonstrated an increase in staff skills and competence to assess swallowing and hydration status, and changes became embedded in practice.

Following care bundle implementation, measures of hydration ranged from 70% (preintervention) to 84% (postintervention), with 73% of participants having a calculated serum osmolarity postintervention. Swallow screening decreased postimplementation, and the evaluation of swallowing improved as 56% of participants received a swallowing evaluation using a range of consistencies. Our postimplementation swallowing screen and/or evaluation (66%) is also comparable with a recently completed European study that was aimed at improving swallowing management in 67 hospitals and achieved 67% adherence to swallowing management practices [29].

Successful implementation was due to several factors. The sites modified whole healthcare systems through interdisciplinary agreements between senior nurses, who approved the reorganisation of infrastructure and processes. This facilitated enhanced nursing practice and resulted in an increased number of patients receiving an evaluation and subsequent management of their swallowing and hydration status. This approach is supported by Pandian et al. [5], who recognise that reorganising existing hospital infrastructure by training healthcare staff to implement simple interventions, such as swallowing evaluations and protocol‐driven care, can be a key element in improving postdischarge morbidity and mortality in LMICs. This codesigned swallowing and hydration care bundle, training of clinical champions, supportive implementation and the modification of management structures resulted in increased staff skills and competence. This contributed to successful implementation of context‐specific care bundle that became embedded in clinical practice.

Experienced‐based codesign is not widely utilised in LMICs. Working in partnership with stakeholders in India (staff, patients and relatives) provided an understanding of the patient pathway, the professional context and local organisation. These insights ensured our research was relevant, addressed areas which stakeholders perceived to need improvement and were considered clinically important.

Codesign activities [30] were key to understanding the value of participatory design, establishing rules for consultation becoming research and recognition of individuals′ commitment to the study. We supplemented the Medical Research Council ‘development, piloting and feasibility, evaluation, reporting and implementation’ model [31] with ‘clinical champions’ (senior nurses) who provided clinical leadership and supported clinicians throughout the day. A train‐the‐trainer model (including training and competency assessments) ensured that the interventions developed were cascaded to new staff members, resulting in the care bundle being sustainably adopted, and nursing staff were confident to undertake the assessments.

The strengths of the study include the use of an experience‐based codesign approach. Similar to other studies [32], this approach was effective in engaging key stakeholders, developing interventions, reorganising processes and facilitating the delivery of interventions for improved stroke care in three acute stroke units across India. Swallowing and hydration interventions were evidence‐based, aligned to global frameworks, culturally sensitive and designed to benefit as many patients as possible. Longer term uptake and sustainability has been achieved. The care bundle has been included as an essential component of stroke unit accreditation by the National Accreditation Board for Hospitals and Healthcare Providers, India.

Limitations, including barriers to implementation included a lack of clarity around staff roles, challenges attending training, lack of availability of equipment, appropriate food and drink consistencies, availability of utensils (e.g., spoons) and carer instructions and training. Similar barriers have been previously reported [33], and, although some barriers may be addressed in the future, others (workforce capacity to attend training and lack of resources) may require different strategies and/or funding.

## 8. Conclusion

We demonstrated that codevelopment and implementation of a low‐cost intervention (a swallowing and hydration care bundle) with appropriate training and competence assessments increased the number of patients who received an evaluation of swallowing and hydration. The care bundle provided an increased odds of a swallowing evaluation of four times higher in participants in the care bundle relative to participants preimplementation (95% confidence interval 2.15 to 7.64 times higher).

Codesign, supportive implementation and modification of management structures were integral to clinical change and ongoing implementation. Experience‐based codesign can be used to underpin the development of context‐specific interventions, increasing the likelihood of sustained implementation. Further research is needed to explore the impact of the care bundle on patient outcomes, the fidelity of future implementation and its applicability in other healthcare systems.

## Funding

This study was supported by National Institute for Health and Care Research (10.13039/501100000272; 16/137/16).

## Conflicts of Interest

The authors declare no conflicts of interest.

## Data Availability

The data that support the findings of this study are available from the corresponding author upon reasonable request.
